# Innovative Strategies and Methodologies in Antimicrobial Peptide Design

**DOI:** 10.3390/jfb15110320

**Published:** 2024-10-29

**Authors:** Devesh Pratap Verma, Amit Kumar Tripathi, Ashwani Kumar Thakur

**Affiliations:** 1 Department of Biological Sciences & Bioengineering, Indian Institute of Technology, Kanpur 208016, Uttar Pradesh, India; 2 Department of Microbiology, Immunology and Genetics, University of North Texas Health Science Center, Fort Worth, TX 76107, USA

**Keywords:** antimicrobial resistance, antibiotics, antimicrobial peptides, peptide drug, host defense, bacterial infection

## Abstract

Multiple lines of research have led to the hypothesis that antimicrobial peptides (AMPs) are an important component of the innate immune response, playing a vital role in the defense against a wide range of infectious diseases. In this review, we explore the occurrence and availability of antimicrobial proteins and peptides across various species, highlighting their natural abundance and evolutionary significance. The design of AMPs has been driven by the identification of key structural and functional features, which are essential for optimizing their antimicrobial activity and reducing toxicity to host cells. We discuss various approaches, including rational design, high-throughput screening, and computational modeling, that have been employed to develop novel AMPs with enhanced efficacy. A particular focus is given to the identification and characterization of peptide fragments derived from naturally occurring host defense proteins, which offer a promising avenue for the discovery of new AMPs. The incorporation of artificial intelligence (AI) and machine learning (ML) tools into AMP research has further accelerated the identification, optimization, and application of these peptides. This review also discusses the current status and therapeutic potential of AMPs, emphasizing their role in addressing the growing issue of antibiotic resistance. The conclusion highlights the importance of continued research and innovation in AMP development to fully harness their potential as next-generation antimicrobial agents.

## 1. Introduction

Penicillin, discovered by Scottish physician and scientist “Alexander Fleming” in 1928, got a Nobel Prize in Physiology/Medicine in 1945. This amazing discovery was not a discovery but an accident or serendipity. Fleming himself wrote the line, “*I did not invent penicillin. Nature did that. I only discovered it by accident*” [1]. This accidentally discovered antibiotic has saved millions of lives from infection worldwide. However, after some time, bacterial pathogens started developing resistance against penicillin. Using similar discovery and production techniques, scientists continuously discovered many other new antibiotics, such as streptomycin, chloramphenicol, erythromycin, vancomycin, and others, to treat the deadly infections caused by various bacterial pathogens [2,3]. Even though the spread of antibiotic-resistant bacteria substantially threatens morbidity and mortality worldwide, the progression of new antibiotic development is challenging.

This phenomenon of antimicrobial resistance (AMR) not only causes death and disability but also imposes significant economic burdens [4,5,6]. Thus, there is an urgent need to improve the treatment of infections caused by resistant pathogens.

**Figure 1:**
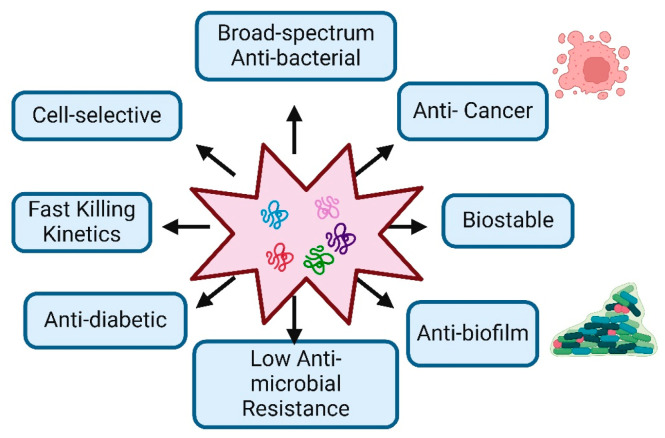
Characteristic Features and Therapeutic Potential of Antimicrobial Peptides: The figure demonstrates the key biological and therapeutic properties of antimicrobial peptides, highlighting their potential applications in medicine.

AMPs are usually compact peptide molecules found in nature, from bacteria to plants, vertebrates, and invertebrates, usually composed of 10 to 50 amino acids. They generally possess a positive charge under physiological pH conditions, which arises from including basic amino acids. This positive charge enables AMPs to engage in electrostatic interactions with the negatively charged bacterial membranes [7]. In comparison to conventional antibiotics, AMPs have a broader range of antibacterial activity against a wide variety of bacteria, fungi, protozoans, viruses, and surprisingly even cancerous cells, compared to traditional antibiotics. They can target a wider variety of bacterial strains, including both Gram-positive and Gram-negative bacteria [8]. Conventional antibiotics typically target specific bacterial components or functions, which can lead to the development of resistance. In contrast, peptides and amino acid-based conjugates often operate through more diverse and less specific mechanisms. They may disrupt various aspects of bacterial physiology, including cell membrane integrity, DNA replication, and protein synthesis, making it more difficult for bacteria to develop resistance. Examples of such peptides include magainin, LL-37 (Cathelicidin), defensin (Human Beta-Defensin-1, HBD-1), and melittin, which are well-known antimicrobial peptides (AMPs) that disrupt the integrity of bacterial membranes [9]. In contrast, peptides such as PR-39, Indolicidin, and Buforin II enter bacterial cells and bind to DNA, inhibiting both DNA replication and transcription without causing significant membrane disruption. Additionally, peptides like Bac7, Pyrrhocoricin, Ocellatin, and Apidaecin inhibit bacterial protein synthesis by binding to ribosomes and interfering with translation [10]. Additionally, antimicrobial peptides (AMPs) can offer benefits beyond bacterial killing, such as immunomodulatory effects and support for tissue repair and wound healing [11]. These benefits position antimicrobial peptides as promising candidates for developing novel antibacterial treatments in the ongoing fight against bacterial infections (Figure 1). This review provides anintroduction to antimicrobial peptides and outlines various strategies for creating peptides with high therapeutic index values. This information could be beneficial to understanding and designing new antimicrobial peptides to address the challenge of bacterial resistance.

## 2. Occurrence and Availability of Antimicrobial Proteins and Peptides

According to a January 2024 report from the Antimicrobial Peptide Database (APD), the database houses a comprehensive collection of approximately 3940 peptides. This includes around 3146 natural antimicrobial peptides (AMPs) spanning six kingdoms: 383 bacteriocins and peptide antibiotics from bacteria, 5 from archaea, 8 from protists, 29 from fungi, 250 from plants, and 2463 from animals. Additionally, the database features 190 predicted antimicrobial peptides and 314 synthetic AMPs (Table 1).

Plants utilize a variety of sophisticated defense mechanisms, including physical and chemical barriers, to protect themselves from a range of infectious organisms such as bacteria, fungi, protozoans, viruses, and insects. These defense mechanisms make plants a promising source of AMPs, which exhibit significant antimicrobial activity against both human and plant pathogens. AMPs can be found in various plant parts, including leaves, roots, seeds, flowers, and stems. As resistance to conventional antibiotics becomes more prevalent, plant-derived antimicrobial peptides continue to offer effective treatment options for severe human diseases.

In animals, a diverse array of cationic proteins with antibacterial properties is produced. These proteins feature cationic peptide sequences that contribute to their antimicrobial activity. Among these, the defensin and cathelicidin families are particularly significant. Cathelicidins are positively charged and amphipathic, varying in size and structure, while defensins, which are crucial for animal immunity, are classified into α-defensins, β-defensins, and θ-defensins based on their disulfide bond arrangements [12].

The following table represents the sources of antimicrobial peptides, their sub-categories, approximate numbers, and a few examples with their biological properties.

**Table 1:** Overview of sources of antimicrobial peptides (reference-Antimicrobial Peptide Database).

Sources	Sub-Category	Approx. Number of AMPs	Examples	Ref.
Plants	Bryophyta to Angiosperms	250	Plant defensins, cyclotides, 2S albumin, lipid transfer proteins, hevein-like proteins knotins, snakins, purothionins	[13]
Animals	Mammals	373	Cathelicidins, Defensins, Bactenecin, Indolicidin, LAP, TAP, Dermcidin, Hepcidin 20, LL-37	[14]
	Amphibians	1179	Magainins, Cathelicidin AL, Buforin, Bombinin, Fallaxin, Magainin, Palustrin 3a, Ranateurin, Phyllospetin	[15]
	Fish	146	Pardaxins, misgurin, C, athelicidin BF, Crotamine, Pelovaterin, Omwaprin	[14]
	Reptiles	52	Cathelicidin BF, Crotamine, Omwaprin, Pelovaterin	[16]
	Birds	47	*d*CATH, AvBD1, *ch*CATH-B1, Fowlicidin 1, CHP2	[17,18]
	Mollusca	54	Defensin A, Mytilin-A, Mytilin-G1, Tachyplesin I, Polyphemusin I and II,	[19]
	Protozoa	6	Discodermin A, Polydiscamide-A, Damicornin	[18]
	Arthropoda	619	Cecropin A, ceratotoxin, stomoxyn, spinigerenin, thanatin, heliomicin, gallerimycin, termicin, royalisin, drosomycin, drosocin, metchnikowin, formaecin, lebocin, pyrrhocoricintin, attacins, coleoptericin, diptericin	[20]
Bacteria		383	Lacticin, Nisin, Lactococcin B, Leucocin A, Enterocin A, Pediocin A, Pediocin F, Pediocin PA-1,Mesentericin Y105, Pediocin AcH, Acidophilin, Acidolin, Lactacin B, Lactacin F, Lactobacillin, Lactobrevin, Reuterin, Plantaricin A, Plantaricin B, Lactolin, Helveticin J	[14,21,22]
Synthetic		314	AMP72, AMP126, AMP2041, BP100, C16, CAMEL0, Dhvar1, Dhvar2, Dhvar4, Dhvar5, FL9, GS14K4, P-Der, Pexiganan, RW BP100, RN7-IN6, WLBU2, WMR-NH2, Pep19-4LF, Guavanin 2	[23]
Fungi		29	defensins, Mytilins, Myticins, and Mytimycin, Dermaseptins	[24]
Predicted		190	GL-29,	[25]
Milk		100	β-lactoglobulin, αs2-casein, β-casein	[26]

## 3. Important Features Considered in Antimicrobial Peptide Design

### 3.1. Length of Amino Acids Within Peptide

The length of antimicrobial peptides is a fundamental criterion for their antimicrobial properties and hemolytic activity. It has been identified that shorter AMPs tend to possess lower levels of hemolytic properties [27]. Consequently, several research efforts are ongoing to remove less important regions of amino acids in the peptide sequence to enhance their biological properties, reduce toxicity, and lower production costs. For example, Kwon et al. designed truncated peptides with shorter lengths that retained antimicrobial activity and exhibited lower toxicity at high concentrations compared to the parent peptide P5. These truncated peptides demonstrated reduced hemolytic activity and fewer toxic effects on mammalian cells compared to P5 [28].

### 3.2. Number of Charged Amino Acids and Their Positions Within the Peptide Chain

The overall charge of the antimicrobial peptides influences their activity and their interaction with the negatively charged bacterial membrane. The interaction between the peptides with the negatively charged phosphate groups on microbial membranes is mainly due to the electrostatic interactions. There are various mechanisms for disrupting the bacterial cell membrane, such as the barrel-stave model, the toroidal model, the carpet model, and the detergent-like model [29,30,31]. The mechanism by which an antimicrobial peptide disrupts bacterial membranes can vary based on the peptide concentration, environmental conditions, and the composition of lipids [31]. Motifs and amino acid substitutions have been seen to play a pivotal role in the design of antimicrobial peptides by directly shaping the peptide’s structure, function, and specificity [32,33]. Motifs, which are recurring amino acid patterns within a peptide sequence, often govern an AMP’s ability to interact with microbial membranes or target specific pathogens. By identifying and incorporating these motifs, AMPs can be designed to mimic the natural defense mechanisms of organisms. For example, the GXXXXG motif in the cytotoxic antimicrobial peptide chrysophsin-1 has been systematically substituted with proline to create non-toxic analogs that maintain similar biological activity but exhibit reduced hemolytic and cytotoxic effects [34]. Amino acid substitutions further refine this design by fine-tuning peptide properties. For instance, replacing bulky hydrophobic residues like phenylalanine can enhance membrane affinity and disruption, while adding cationic residues strengthens electrostatic interactions with negatively charged microbial membranes [35,36,37].

The antimicrobial effectiveness of AMPs is significantly boosted by their positive charge, especially from Arginine (Arg) and Lysine (Lys) residues, which interact with the highly negatively charged microbial membrane. These positive residues are essential in designing new AMPs to enhance antimicrobial activity and selectivity [38,39].

Research has also shown how the distribution of positive charges affects the antimicrobial and hemolytic properties of AMPs. By replacing particular amino acids in AMPs with positively charged residues, such as lysine, scientists have observed a decrease in cytotoxicity and hemolytic activity. This modification helps make these peptides safer for therapeutic use, as it enhances their ability to target and eliminate microbial cells while minimizing damage to human red blood cells [40,41,42].

### 3.3. Percent Hydrophobicity and Amphipathicity

Hydrophobicity is the most important feature of antimicrobial peptides. It is responsible for the interaction of hydrophobic amino acids with the fatty acid acyl chain of the lipid membranes of bacteria. That helps the insertion of the transmembrane portion of the peptide with the hydrophobic core of the bilayer. The most frequent hydrophobicity percentages observed were 43% and 44%. Consequently, the primary criteria for designing new AMPs were established: a peptide length of 13 or 14 amino acids, a +4 charge, and 43–44% hydrophobicity. Therefore, the designed AMP sequence should include 5 hydrophobic residues for a 13-residue-long AMP and 6 hydrophobic residues for a 14-residue-long AMP [43].

Generally, moderately hydrophobic peptides show optimal activity, whereas highly hydrophobic peptides tend to exhibit strong hemolytic activity and reduced antimicrobial effectiveness [44].

Amphipathicity, which refers to the distribution of hydrophilic and hydrophobic residues on opposite faces of peptides, significantly influences the binding affinity of α-helix AMPs to membranes. In amphipathic AMPs, the hydrophobic residues attach to the lipid bilayer, while the hydrophilic residues interact with phospholipid groups [45].

## 4. AMPs Derived from Naturally Occurring Proteins

**Figure 2:**
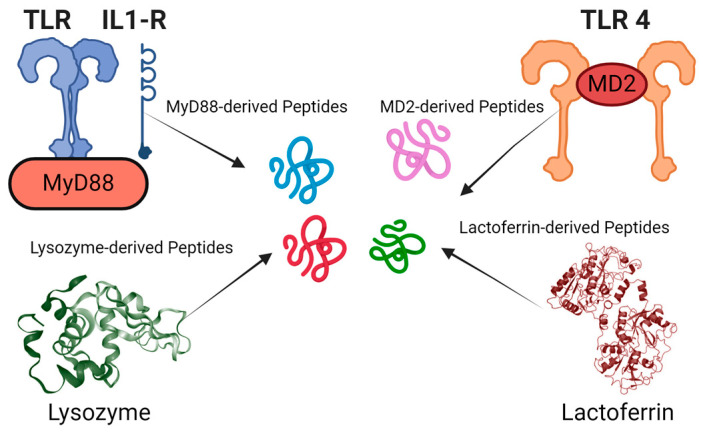
Identification of antimicrobial and immunomodulatory peptide fragments from various protein sources: The figure showcases peptide fragments derived from proteins such as lysozyme, MyD88, MD2, and lactoferrin. These examples show the diverse origins of peptides with potential antimicrobial and immunomodulatory activities.

AMPs derived from naturally occurring proteins can act as negative regulators by exploiting structural and functional similarities with their parent proteins. These peptides often mimic key regions of the parent protein, allowing them to bind to the same targets or interact with the same molecules. However, rather than promoting the protein’s normal function, they can inhibit it by competing for binding sites, preventing the parent protein from interacting with its natural ligands or substrates. In some cases, AMPs may bind to allosteric sites, inducing conformational changes that impair the protein’s functionality, such as disrupting an enzyme’s active site or destabilizing its structure. Additionally, they can interfere with protein-protein interactions, blocking the formation of functional complexes required for the parent protein’s activity. This leads to a reduction or inhibition of the protein’s normal function, essentially acting as a “negative” version of the parent protein. In some cases, these peptides may even cause the formation of non-functional aggregates, further disabling the protein. This ability to selectively inhibit or modulate the activity of their parent proteins offers therapeutic potential, particularly in conditions where reducing specific protein activity is beneficial. Researchers typically begin by analyzing the sequences and structures of natural proteins and AMPs, such as those derived from host defense proteins in various organisms (Figure 2).

Some natural peptides have also served as templates for rational design strategies, which may include modifying amino acid compositions, adjusting peptide lengths, or introducing chemical modifications to enhance antimicrobial activity and stability. Common methods involve truncating existing peptide sequences, introducing mutations, cyclization, and incorporating unnatural amino acids. Examples of biologically active peptides derived from naturally occurring proteins include synthetic AMPs and immunomodulatory peptides that have been developed from proteins like Myd88 and MD2, demonstrating effectiveness in neutralizing the lipopolysaccharides of Gram-negative bacteria and protecting mice from endotoxin-mediated lung infection and death [46,47,48]. This approach has also been applied to design anti-cancer peptides from proteins like MIEN1 and Annexin A2, which are specific to cancer cells [49,50,51]. Since anticancer and antimicrobial peptides share similar biophysical parameters, this approach can also be applied to the design of antimicrobial peptides.

### 4.1. Identification and Characterization of Numerous Peptide Fragments from Naturally Occurring Host Defense Proteins

The identification and characterization of peptide fragments derived from naturally occurring host defense proteins has been an active area of research in antimicrobial peptide discovery. Numerous studies have isolated bioactive peptide fragments from larger host defense proteins and characterized their antimicrobial properties. For example, lactoferricin is a potent antimicrobial peptide fragment derived from the milk protein lactoferrin. Similarly, fragments of human cathelicidin LL-37 have been shown to retain antimicrobial activity while reducing toxicity. Lactoferricin, the 25-amino acid peptide from lactoferrin, exhibits broad-spectrum activity against bacteria, fungi, and viruses. Human cathelicidin LL-37 has yielded several active fragments, including KR-12, FK-13, and RK-31, each with distinct antimicrobial properties. Lysozyme-derived peptides like LzP and HEL96-116 show activity against various microbes. Thrombin has produced GKY25, a fragment with broad-spectrum antimicrobial activity. Defensins have yielded active fragments from HNP-1 and HD5. Buforin II, a 21-amino acid peptide from histone H2A, can penetrate cell membranes and bind to nucleic acids. Indolicidin, the widely studied 13-amino acid peptide from bovine neutrophils, is known for its unique linear structure and membrane permeabilization properties. PR-39, derived from porcine cathelicidin, inhibits bacterial protein and DNA synthesis. Cecropin A, a 37-amino acid peptide from the moth Hyalophora cecropia, forms pores in bacterial membranes. Dermaseptin S4, from the South American frog *Phyllomedusa sauvagii,* exhibits broad-spectrum activity against bacteria, fungi, and protozoa. These diverse peptide fragments often demonstrate enhanced antimicrobial potency, reduced toxicity, and improved stability compared to the parent protein.

One of the key factors in identifying these peptide fragments lies in understanding their amino acid composition, charge distribution, and hydrophobicity. These characteristics play a crucial role in determining the effectiveness of the peptides against bacterial targets. Studies have shown that a careful balance of approximately 40% hydrophobicity and 60% charged residues enhances the likelihood of these peptides exhibiting potent antibacterial properties [44].

### 4.2. Denovo Approach to the Design of Short AMPs Based on the Sequence Pattern of Naturally Occurring Antimicrobial Proteins

Drawing inspiration from these naturally occurring peptides, such as those found in the cathelicidin family, researchers can identify them using common patterns, often characterized by the presence of cationic residues at the N-terminus and negatively charged residues elsewhere. Leveraging this understanding, scientists have devised designer peptides adhering to similar structural criteria.

These designer peptides are meticulously engineered, often utilizing helical wheel diagrams to ensure an amphipathic nature [52,53]. Several de novo-designed short antimicrobial peptides have been developed based on sequence patterns of natural antimicrobial proteins. These include pexiganan, a 22-amino acid analog of magainin with broad-spectrum activity, and its optimized version, MSI-78 (pexiganan acetate), with improved potency and reduced hemolytic activity. SMAP-29, a 29-amino acid peptide based on cathelicidins, features a cationic N-terminus and amphipathic structure [54]. P-113, derived from histatin 5, is a 12-amino acid peptide designed for retained activity with improved stability. Temporin-1CEa, a synthetic 13-amino acid peptide based on temporins, shows enhanced antimicrobial activity and reduced hemolysis. Melimine, a 29-amino acid chimeric peptide combining elements from melittin and protamine, was designed for improved activity and reduced toxicity. WLBU2, a 24-amino acid-engineered cationic peptide, forms an idealized amphipathic helix with broad-spectrum activity [55]. Omiganan, a 12-amino acid synthetic peptide based on indolicidin, is active against various pathogens. LL-37 analogs mimic the properties of human cathelicidin LL-37 with improved stability and reduced toxicity. The hybrid peptide such as cecropin A-melittin combines elements from both peptides to optimize antimicrobial activity while minimizing cytotoxicity.

### 4.3. Designing of AMPs Using Bioinformatics Tools

The advent of advanced computer-based techniques has significantly accelerated the in silico design and development of novel designer peptides and proteins as new therapeutics. Peptide and protein databases have emerged as practical computational tools in drug discovery, providing comprehensive sequences and structures with diverse bioactivities. This process utilizes computational tools and algorithms to predict the structure-activity relationship of target peptides by analyzing properties unique to AMPs, followed by assessing their potential antimicrobial activities [25].

There are several specialized antimicrobial peptide databases available to aid in peptide design. The Antimicrobial Peptide Database (APD) is the most important computational tool that facilitates the comprehensive search, analysis, and design of antimicrobial peptides [56]. Likewise, the Collection of Anti-Microbial Peptides (CAMP) houses a vast array of sequences, structures, and family-specific signatures of antimicrobial peptides, serving as a critical repository for antimicrobial research [57]. Another significant resource is the Database of Anuran Defense Peptides (DADP), which includes numerous entries with some containing minimum inhibiting concentration (MIC) data against microorganisms [58].

Computational approaches for designing novel antimicrobial peptides often rely on structure-activity relationship (SAR) analysis.

In addition to predicting biological activity, online services also provide structural and conformational information about peptides. Tools such as Helical Wheel Projections help visualize alpha-helical structures and the arrangement of hydrophobic and hydrophilic residues within peptides [59]. These visualization tools are essential for optimizing the design of effective antimicrobial peptides, ensuring that the peptides possess the desired structural characteristics for their intended biological activity. The Data Repository of Antimicrobial Peptides (DRAMP) is a more recent and comprehensive database, containing 30,260 entries as of its latest update (version 4.0). DRAMP includes general AMPs, patent sequences, and clinical peptides, providing information on sequences, structures, activities, physicochemical properties, patents, clinical trials, and references [60]. It also includes new annotations on serum stability and protease stability of AMPs, crucial for clinical translation. Another important tool is the Database of Antimicrobial Activity and Structure of Peptides (DBAASP). It is a manually curated, open-access resource for antimicrobial peptides (AMPs) that contains detailed information on over 15,700 peptides, including their sequences, chemical modifications, 3D structures, antimicrobial activities, and toxicities. DBAASP also covers ribosomal, non-ribosomal, and synthetic peptides, providing data on their activities against more than 4200 specific target microbes [61].

Several AMP prediction tools utilize deep learning approaches beyond the well-known AMPScanner, notable examples of which include AMPlify, which employs an attentive deep learning model using bidirectional long short-term memory (Bi-LSTM) and attention mechanisms; Deep-AmPEP30, which utilizes a convolutional neural network (CNN) for predicting AMPs but is limited to peptides up to 30 amino acids in length [62]; Deep-ABPpred, which combines Bi-LSTM with word2vec for AMP prediction; sAMPpred-GAT, which implements Graph Attention Neural Networks (GAT) for AMP prediction; and AMPs-Net, which uses Graph Convolutional Networks (GCN) to predict AMP characteristics [25]. Recent developments addressing non-linear peptides include LABAMPsGCN, which employs Graph Convolutional Networks and Chebyshev Spectral CNN, and geometric deep learning approaches, which are suited for representing the three-dimensional structure of peptides, including non-linear ones [63].

### 4.4. ML/AI-Guided Approaches

**Figure 3:**
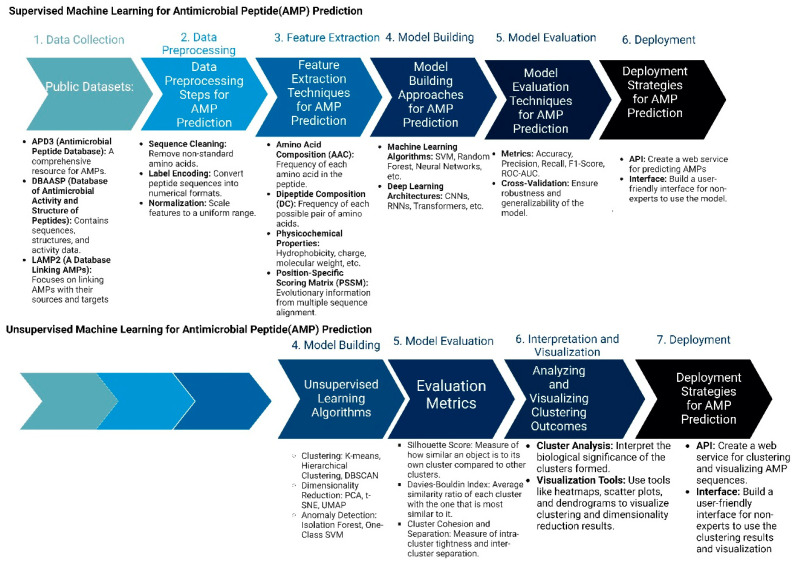
Machine Learning Approaches in Antimicrobial Peptide Design: Supervised vs. Unsupervised Methods: The figure compares supervised and unsupervised machine learning.

In drug discovery, AI (artificial intelligence) and ML (machine learning) are being developed as new models to design new antimicrobial peptide therapies (Figure 3). Recent developments in artificial intelligence (AI) and machine learning (ML) have revolutionized our capacity to forecast biomolecular properties and structures, as well as to design new molecules. Utilizing machine learning for peptide modeling has the potential to address many of the limitations of conventional drug discovery, thereby facilitating the swift development and clinical application of AMPs [64,65].

Two important methods of peptide design include supervised and unsupervised machine learning methods. In supervised learning approaches, the process begins with the meticulous collection of comprehensive datasets that include known AMPs along with their recorded activities, as well as negative examples of non-effective peptides. These peptide sequences are then preprocessed and transformed into numerical formats, such as one-hot encoded vectors or embeddings, to facilitate machine learning analysis. Feature extraction follows, where critical properties such as amino acid composition, hydrophobicity, and charge are quantified. With these features, researchers select suitable machine learning algorithms, like Random Forests or Neural Networks, and train them using the processed data. The performance of these models is rigorously evaluated through various metrics, such as accuracy and precision, to ensure their predictive capability. Once validated, these models are employed to predict the antimicrobial activity of novel peptide sequences or to design new AMPs with desired characteristics. The final step involves experimental validation of the top-ranked peptide predictions to confirm their effectiveness in real-world applications.

In contrast, unsupervised learning methods aim to discover patterns within AMP sequences without relying on labeled activity data. This process also starts with data collection and preprocessing but shifts focus to exploratory data analysis. Techniques such as dimensionality reduction (e.g., Principal Component Analysis) are used to visualize and understand the structure of the AMP sequence space. Clustering algorithms, such as K-means or hierarchical clustering, are then applied to group similar AMPs based on their sequence and property similarities. Pattern discovery techniques help identify common motifs or structural features within these clusters. A pivotal aspect of unsupervised learning in AMP design is the use of generative models, like Variational Autoencoders (VAEs) or Generative Adversarial Networks (GANs). These models learn the underlying distribution of AMP sequences and can generate novel, previously unseen peptides. The generated sequences are filtered based on desired properties and evaluated through existing supervised models or expert knowledge. Promising candidates are synthesized and experimentally tested, and the results are used to refine and improve future models. This iterative cycle of generation, evaluation, and refinement facilitates the continuous exploration and expansion of the AMP design space, potentially leading to the discovery of highly effective and innovative antimicrobial peptides.

### 4.5. Genome Mining Approaches from Natural Environments

Genome mining approaches from natural environments have emerged as a powerful tool for discovering novel antimicrobial peptides (AMPs). This method leverages the vast genetic diversity found in microorganisms and other organisms in their natural habitats to identify potential AMP-encoding genes. Researchers use bioinformatics tools to analyze genomic and metagenomic data, searching for sequences that share similarities with known AMPs or possess characteristics typical of antimicrobial peptides. This approach has led to the discovery of several promising AMPs from diverse sources.

One notable example is the identification of blenniorphins, a group of peptides derived from blenny fish [66]. These peptides were discovered through a genome mining approach that searched for sequences homologous to the endogenous kappa opioid receptor peptide dynorphin A. The resulting blenniorphins demonstrated nanomolar affinity and potency at the kappa opioid receptor, highlighting the potential of fish-derived peptides as a source of novel ligands.

Another example is the discovery of lasso peptides through genome mining [67]. Researchers have developed specialized tools and algorithms to identify biosynthetic gene clusters encoding these unique peptides in bacterial genomes. This approach has led to the identification of numerous novel lasso peptides with potential antimicrobial activities.

Additionally, genome mining has been used to identify novel lantibiotics, a class of ribosomally synthesized and post-translationally modified peptides with antimicrobial properties [68]. Tools like RiPPMiner and PRISM have been instrumental in predicting and characterizing these peptides from genomic data.

These examples demonstrate the power of genome mining approaches in natural environments for designing and discovering new antimicrobial peptides, expanding our arsenal of potential therapeutic agents against drug-resistant pathogens. The detailed discovery of new antimicrobial peptides from cone snails using a combined proteo-transcriptomic approach has been shown using genome mining approaches [69].

A study by Zhu et al. provides a proof of concept that mining the microbial genome is a promising approach to discovering efficient AMPs for systemic therapy of bacterial infectious diseases [70].

## 5. Engineering of Peptide Fragments to Enhance Their Therapeutic Index

AMPs, whether naturally occurring or intentionally designed, can be enhanced to boost their antimicrobial properties, cell selectivity, biological stability, and other attributes. Strategies to improve these characteristics include modifying hydrophobicity, hydrophilicity, charges, and cleavage sites within the peptide sequence. For instance, chemical alterations and the incorporation of non-standard amino acids/chemical moieties can significantly enhance antimicrobial peptides for greater therapeutic efficacy. Optimization techniques such as adjusting hydrophobic interactions can influence how peptides interact with lipid membranes or specific targets while increasing hydrophilicity can improve solubility and reduce aggregation (Table 2). Additionally, controlling peptide charges by modifying charged amino acids affects interactions with bacterial membranes and mammalian cells. Strategic modification of cleavage sites helps regulate susceptibility to proteolytic degradation, thereby improving peptide stability and lifespan. These approaches collectively offer promising pathways to overcome existing limitations in peptide-based therapies, potentially enhancing their performance and effectiveness in treating various health conditions.

## 6. Synergistic Approaches to Develop Antimicrobial Peptides with Conventional Antibiotics

**Figure 4:**
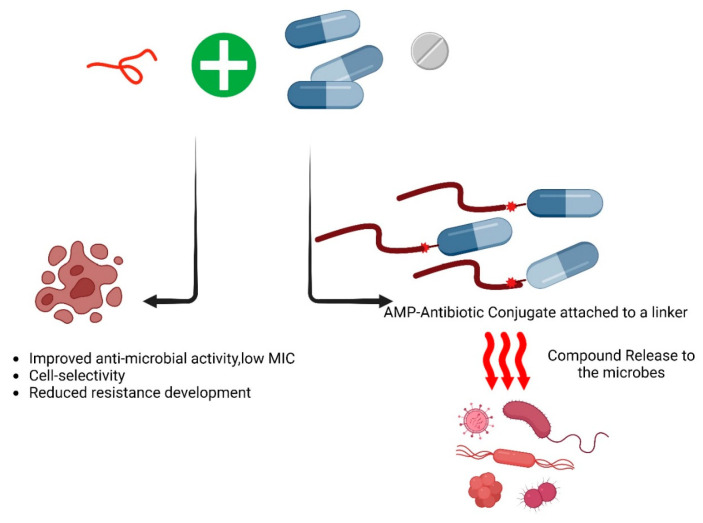
The combinational effect of antimicrobial peptides with conventional antibiotics. As of now, based on the AMPs database, 47 of the listed antimicrobial peptides (AMPs) demonstrate synergistic activities against various pathogens. Exploring this synergistic or additive effect is valuable for identifying novel and effective therapeutic approaches against the ESKAPE pathogens. Following is the list of the antimicrobial peptides that showed synergy with themselves and/or other antimicrobial agents such as antibiotics [89].

Developing new antibiotics is a complex and costly process, making the repurposing of existing antibiotics with modifications a more practical approach. Several strategies have been developed to enhance conventional antibiotics, one of which is combination therapy (Table 3). This approach involves pairing antimicrobial agents with antibiotics to improve their efficacy and overcome resistance [86]. It has been identified that the Fmoc-Phe showed a synergistic effect with Aztreonam and increased its efficacy by reducing its MIC by 60-fold against Gram-negative bacteria [82]. Combination therapies are employed to enhance the effectiveness, stability, and cell selectivity of both antibiotics and peptides, especially when peptide-to-peptide synergism is observed, while also reducing the likelihood of resistance development in pathogens [87,88]. Ongoing research is exploring the synergistic effects of various antibiotic concentrations. These combinatorial approaches hold the potential for developing novel antimicrobials with high therapeutic efficacy in the future (Figure 4).

The synergistic effects of the antimicrobial peptides with the antibiotics are calculated using adding the fractional inhibitory concentration (FIC) of the peptide with the FIC of antibiotics. The FIC index for the determination of combinational effect is calculated using the following formula:

FICI (peptide/antibiotic combination) = (MIC of peptide in the presence of antibiotics/MIC of peptides alone) + (MIC of antibiotic in the presence of peptides/MIC of antibiotic alone) if FIC ≤ 0.5, it indicated synergy; if FIC ≤ 4, it indicated an additive effect; and if FIC > 4, it indicated antagonistic actions. The FIC index was calculated by summing the FIC values of the antibiotics and peptides.

## 7. Amino Acid-Based Conjugated Antimicrobial Agents

Structurally, amino acids consist of an amine (-NH2) and carboxylic (-COOH) functional group, along with a side (R) group that varies in length, determining the physiological properties of proteins and peptides based on their hydrophobic, hydrophilic, polar, or non-polar nature. Similarly, antimicrobial peptides and amino acid conjugates exhibit varying degrees of potency in inhibiting or killing bacteria through mechanisms such as cell membrane disruption or penetration. It is anticipated that antimicrobial drugs conjugated with amino acids could yield novel leads with promising pharmacological activities. Amino acid conjugates are formed by chemical bonds between various hydrophobic and hydrophilic groups and various amino acids, particularly those exhibiting antibacterial activity against infectious bacteria. Numerous amino acid–natural compound conjugates have shown improved pharmacokinetic characteristics, including absorption, distribution properties, reduced toxicity, and increased physiological effects [90]. The simplicity of manipulating amino acids enables targeted pharmacological activities, with several conjugates reported involving compounds like curcumin, astaxanthin, and quercetin [91]. The conjugations aim to enhance pharmacological activities, decrease toxicity, improve target specificity, and increase absorption through peptide transporters.

## 8. Current Status and Application of AMPs

It is now well established that antimicrobial peptides, a diverse group of molecules derived from various sources, possess extensive antimicrobial, immunomodulatory, and host-beneficial activities. These include anticancer effects and wound-healing properties, highlighting their broad therapeutic potential [92]. They are particularly effective against multidrug-resistant bacteria, with the advantage that resistance to AMPs is rarely developed, underscoring their promising therapeutic potential. A few AMPs have already been approved by the US Food and Drug Administration (FDA) for clinical use. For instance, Gramicidin, sourced from Bacillus brevis, disrupts bacterial cell membranes and is particularly potent against Gram-positive bacteria, also showing inhibitory effects on certain Gram-negative bacteria at higher concentrations [93]. Noteworthy FDA-approved AMPs available commercially, including Polymyxin B and Daptomycin, are used to treat skin infections.

Despite the progress, most AMPs are still in the preclinical phase, with only a few advancing to clinical trials. Table 4 provides an overview of AMPs currently undergoing clinical or preclinical evaluation. One example is EA-230. It is a synthetic tetrapeptide from human chorionic gonadotropin and shows promise as a therapeutic agent with immunomodulatory and anti-inflammatory properties. In a phase 2 trial involving patients with cardiac surgery, it was safe and well-tolerated, improving renal function and fluid balance, though it did not significantly affect interleukin-6 levels [94].

Continued research and clinical trials are essential to enhance peptide stability, minimize cytotoxicity, and improve their pharmacokinetic and pharmacodynamic profiles for better clinical application [95].

**Table 4:** Some selected AMPs in Different Stages of Clinical Trials. The table is adapted from the work of Xin Li et al. [96].

AMPs	Origin	Clinical Properties	Mechanism of Action	Clinical Phase	Ref.
EA-230	human chorionic gonadotropin	Phase 1/2	Intravenous	immunomodulatory and renoprotective effects	[94]
Iseganan	Protegrin-1	Phase 2/3	Topical	Prevention of ventilator-associated pneumonia	[97]
XF-73	Porphyrin	Phase 1	Nasal gel	Prevention of postoperative *S. aureus* colonization and infection	[98]
P-113	Histatin 5	Phase 2	Mouth rinse	Reduce gum bleeding, gingivitis, and plaque	[99]
Omiganan	Indolicidin	Phase 2	Topical gel	Treatment of mild to moderate atopic dermatitis	[100]
LTX-109	Synthetic peptidomimetic	Phase 1/2	Topical	Prevention of nasal infections caused by methicillin-sensitive/resistant *S. aureus*	[101]
Onc72	Oncocin	Preclinical	Subcutaneous	Treatment of antibiotic-susceptible *K. pneumoniae*	[102]
OP-145	LL-37	Preclinical	Implant coating	Prevention of *S. aureus*-induced biomaterial-associated infections	[103]
Lactoferrin	Not applicable	Phase 4	Oral	Prevention of neonatal sepsis	[104]
Murepavadin	Protegrin-1	Phase 1	Intravenous	Treatment of pneumonia caused by *P. aeruginosa* infection	[105]
Surotomycin	Daptomycin	Phase 2	Oral	Treatment of C. difficile-associated infection	[106]
LL-37	Not applicable	Phase 2	Topical	Control of infection of diabetic foot ulcers	[107]

## 9. Conclusions and Future Prospects

Antimicrobial peptides (AMPs) stand at a pivotal moment in the battle against antibiotic resistance, offering immense promise as alternatives to conventional antibiotics. Their rapid killing kinetics and diverse modes of action position them as valuable candidates for combating a wide range of pathogens. However, challenges such as stability, cell selectivity, and bioavailability remain significant obstacles to their clinical application. Moving forward, a multidisciplinary approach is essential. Advances in chemical modifications, bioengineering, and novel delivery systems will be key to addressing the current hurdles faced by AMPs. Integrating computational modeling and high-throughput screening can accelerate the discovery and optimization of new peptide candidates. Furthermore, combining AMPs with conventional antimicrobials could enhance therapeutic efficacy and reduce resistance development, offering a multi-faceted strategy for treatment. As research progresses, maintaining a balance between optimizing efficacy and ensuring safety is critical. The potential for AMPs to revolutionize antimicrobial therapy is significant, but realizing this potential will require sustained innovation, collaboration, and a deep understanding of structure-activity relationships. By harnessing the natural power of AMPs while addressing their inherent limitations, we are moving closer to a new era in antimicrobial therapy, one that holds great promise in the ongoing fight against infectious diseases.

## Figures and Tables

**Table 2:** The different modifications in peptide fragments to enhance their therapeutic potential with some examples.

Modifications	Substitute/Addition Moieties	Peptide	Function	Ref.
N-Acetylation	Acetyl group	maximin H5	Increased activity and stability	[71]
Amidation of C-terminus	Amide group	maximin H5	lower levels of hemolysis,	[72]
Amino acid conversion	Substitution of amino acids, Lys, His, Ser substitution to Arg amino acid residue, Phe replaced to Trp	Pexiganan	Improved antimicrobial activity with enhanced cell selectivity	[72]
Cyclization	cross-linking constructions with disulfide bonds and cyclization by lactam ring	Baciim, Cubicin	Increased permeability, stability, and bioactivity of AMPs	[73]
Fatty acid coupling	decanoic acid, lauric acid, myristic acid	C10-PR-Spn, C12-PR-Spn, C14-PR-Spn	Increased antibacterial activity and stability	[74]
Glycosylation	glycan moiety/sugar moiety		Enhances the antimicrobial properties of AMPs, as well as their stability and biological properties, immunomodulation	[75]
Unusual amino acids	Lanthionine, 3-methyllanthionine, and Dehydrobutyrine, D-form of amino acids	Nukacin ISK-1, Chem-8dK	Antibacterial Gram-positive, bacteria, cell selectivity	[76,77]
Disulfide bonds	The bond between two cysteine amino acids	Thanatin, β-defensin 3	Stabilized structure	[78,79,80]
Hydrogel	aztreonam encapsulated Fmoc-F hydrogels	Fmoc-F	Increased efficacy and selectivity against bacteria	[81,82]
Halogenation	chlorine, fluorine, bromine, and iodine	Jelleine-I,	Improvement of degradability of therapeutic agents, lipophilicity, catabolic stability, and membrane permeabilization properties	[83,84,85]

**Table 3:** Some examples of AMPs demonstrating synergistic effects either among themselves or in combination with antibiotics. The table is adapted from the work of Rima et al. [89].

Antimicrobial Peptides (AMPs)	Origin	Synergistic Molecule	Antimicrobial Properties
PGLa	Frog skin	Magainin 2	*E. coli* and *S. aureus*
Ranalexin	Bullfrog R. Catesbeiana, Staphylococcus simulans	Endopeptidase lysostaphin	*S. aureus* (*MRSA*)
P10		Ceftazidim/ doripenem	*MDR A. baumannii* and *colistin-resistant* *P. aeruginosa*
Tridecaptin M	Mud bacterium	Rifampicin, vancomycin, and ceftazidime	drug-resistant *A.* *baumannii*
Dermaseptin	Amphibians skin	Dermaseptin	*E. coli*, *P. aeruginosa*, *S. aureus*
Lactoferricin	Mammalians milk	Ciprofloxacin, ceftazidime	*P. aeruginosa*
Nisin	Lactococcus lactis	Colistin	*Pseudomonas* biofilms
Gad-1	Fish	Kanamycin, ciprofloxacin	*P. aeruginosa*
Bactenecin	Lactic acid bacteria	Bactenecin	*E. coli*, *P. aeruginosa*, *S. Typhimurium*
PMAP-36	Porcine	tetracycline	*Escherichia coli*
AA230	Arenicin-3	EDTA	*Pseudomonas aeruginosa* or *Escherichia coli*
Melimine	Hybrid peptide of melittin and protamine	ciprofloxacin	*P. aeruginosa* 37
